# Translation and cross-cultural adaptation of the National Health Service Sustainability Model to the Chinese healthcare context

**DOI:** 10.1186/s12912-023-01293-x

**Published:** 2023-04-15

**Authors:** Jie Lai, Lynne Maher, Chaixiu Li, Chunlan Zhou, Hasan Alelayan, Jiaqi Fu, Yanni Wu

**Affiliations:** 1 Nanfang Hospital, Southern Medical University, Guangzhou City, Guangdong Province People’s Republic of China; 2 School of Nursing, Southern Medical University, Guangzhou, People’s Republic of China; 3 Ko Awatea I Health System Innovation and Improvement, Middlemore Hospital, 100 Hospital Road, Otahuhu, New Zealand

**Keywords:** Evidence implementation, Evidence-based practice, Sustainability, Cognitive interviewing, Cross-cultural adaptation

## Abstract

**Background:**

International attention is being paid to the issue of making evidence sustainable after implementation. Developing an identification model is essential to promote and monitor the sustainability of evidence implementation. However, this model is not available in Chinese. This study aims to translate the National Health Service Sustainability Model into Chinese and to verify whether the model is adapted to the Chinese healthcare environment.

**Methods:**

This study follows the translation and validation guidelines developed by Sousa and Rojjanasrirat. The translations include forward and backward translations and their comparison. Expert reviews were used to validate the content validity of the Chinese version of the National Health Service sustainability model. Cognitive interviews were used to assess the validity of the language in the Chinese setting.

**Results:**

The translation was conducted by a bilingual research team and took 12 months. Expert reviews were undertaken with eight experts, and cognitive interviews with six participants. The content validity of the model is excellent, but at least 20% of the experts still felt that items one, three, five and nine needed refinements. In the cognitive interviews, most items, instructions and response options were well understood by the participants responsible for the evidence-based practice project. However, some language issues were still identified in items one, three, four, five, seven, nine, and ten. Participants reported that the sustainability results of the model assessment were consistent with their previous judgments of the items. Based on the expert review and interview results, items one, three, four, five, seven, nine and ten require further refinement. In summary, seven of the ten items have been amended.

**Conclusions:**

This study provides insight into how the National Health Service sustainability model can be used in the Chinese healthcare setting and paves the way for future large-scale psychometric testing.

**Supplementary Information:**

The online version contains supplementary material available at 10.1186/s12912-023-01293-x.

## Background

Evidence-based healthcare has been widely recognised as improving healthcare delivery quality and patient experience [1]. Increasingly, evidence-based practice (EBP) or evidence implementation is also being introduced into clinical practice by healthcare organisations [2]. A scoping review published in 2015 showed that the number of EBP projects in nursing practice in China has gradually grown from zero to 28 [3]. As expected, these projects have increased patient satisfaction with care and improved patient clinical outcomes [4–6].

It is recognised that the design of EBP requires a high level of time commitment from staff participants. This is often not built into the clinical practice workload, and as a result, EBPs are not adopted and sustained over time [7]. A previous study identified that quality improvement projects in the UK did perform well during the project's active timescale but were not sustained over time, resulting in wasted upfront investment [8]. A systematic review which included 125 healthcare improvement projects, found that even when full implementation was achieved, most programs did not achieve sustainability of all aspects of the project as initially designed or achieved [9]. Another systematic review focusing on implementing clinical practice guidelines found that about half of the 14 studies reported a decline in compliance with clinical practice guidelines by medical staff, returning to the previous routine [10].

The failure to maintain EBP can result in reduced interest and confidence of medical staff in investing time to introduce evidence into clinical practice. [2]. In a community context, none sustainability of improvements in care pathways may result in a lower level of community support and trust in healthcare organisations [11]. Therefore, it is necessary to identify how to influence the sustainability of implemented initiatives to maintain staff confidence to test and implement improvements and achieve long-term impact through the best use of financial investments [9, 12].

In recent years, a growing body of research has considered the challenges of sustaining evidence-based practice projects [13–15], and many scholars have developed models or frameworks to promote and monitor the sustainability of evidence implementation in healthcare settings [16, 17]. A systematic review identified that despite no clear consensus on how to define or influence sustainability, 62 approaches to assessing healthcare sustainability, including theories, frameworks and models, have been proposed within the literature up to 2017, with an average of two assessment methods created every two years since 1980 [2]. A scoping review of sustainability assessment tools for clinical practice programs published in China in 2021 also noted that China needs a culturally appropriate sustainability assessment tool [18]. Currently, most published sustainability tools are from developed countries [2]. As China is located in East Asia, one of the largest developing countries in the world, its clinical leadership hierarchy is different from that of many developed Western countries. The dominant culture of nursing leadership in Chinese clinics is centralised with the role of the Head Nurse, and most clinical nurses in China have less theoretical knowledge and experience in EBP [19]. A scoping review published in China showed that as of 17 January 2020, there were 152 articles published in Chinese on evidence implementation projects in nursing. Only 7.2% of the articles suggested that evidence should be consistently applied in clinics, and 3.3% indicated specific strategies to maintain sustainability in the clinical setting [20]. This suggests that although evidence implementation projects in nursing have gradually increased over the past 20 years in China, there needs to be more research on evidence translation and sustainability in nursing practice in China [20]. Therefore, to better facilitate the implementation and maintenance of evidence, there is an urgent need to develop or translate a culturally appropriate tool for assessing the sustainability of evidence implementation projects in China [18].

To date, the sustainability approaches often explicitly stated to be applied in clinical practice are the Normalisation Process Theory, the Normalisation Process Model, the Programme Sustainability Assessment Tool and the National Health Service (NHS) Sustainability Model (SM) [21]. The NHS SM is a diagnostic tool developed to help predict the sustainability of organisational change and provide strategies for sustaining change outcomes [22]. The model was developed through a rigorous process, initially incorporating 100 factors that influence sustainability through an extensive collection of literature on sustainability in management and interviews with project leaders, managers, clinical staff, quality control experts, global health experts, and others. The 100 factors were then weighted on a scale of 1 to 10 through focus interviews with 250 internal NHS staff and health management experts who identified from their perspective which of the factors was most relevant and essential. Ten factors emerged through this process and were included in the NHS Sustainability Model [23]. One study notes that it is one of the most comprehensive of the existing sustainability development frameworks and is organised in a checklist format that facilitates knowledge translation [24]. This study aimed to translate the NHS SM into Chinese and to verify that the Chinese version of NHS SM can be adapted to the Chinese healthcare context.

## Methods

### Design of the study

This study adheres to systematic guidelines based on a comprehensive review of methods for translating, adapting, and validating cross-cultural research tools [25]. Before the translation process began, we had obtained permission from the original author of the NHS SM via email to translate the NHS SM into Chinese.

### Step 1: Forward translation

The original English (source language) version of NHS SM was independently translated into Chinese (target language) by two bilingual translators who are native Chinese speakers and have passed the College English Test Band Six in China. One translator has a master's degree in nursing and knows evidence-based nursing terminology. The other is an undergraduate student majoring in international economics and trade with no medical background [25].

### Step 2: Comparison of the two forward translation versions

A forward translation committee compared the two translated versions of NHS SM and examined the differences between words, sentences, and meanings. The committee comprises three Chinese academics, including a bilingual PhD candidate in the UK who is an evidence implementation trainer at the Joanna Briggs Institute (JBI), an undergraduate student majoring in English translation, and a Master of Nursing student. The committee discussed the differences and assessed whether the translations were:

1. Conceptual equivalence: conceptual understanding in Chinese healthcare.
2. Semantic equivalence: correctly reflected the intended meaning in English.
3. Content equivalence: the content of each scale entry is culturally appropriate for the population in which the scale is used.
4. Operational equivalence: having wording, format, instructions and scales that can be used in a Chinese healthcare context.

All discrepancies were resolved through discussion with the committee and the two forward translators together until all reached a consensus. A preliminary Chinese version of the NHS SM was formed at this step.

### Step 3: Blind backward translation

The preliminary Chinese version of the NHS SM was independently back-translated into English by two translators who were native English speakers and had a good command of Chinese. Similarly, one translator with medical background and the other without a medical background, and neither translator had been previously exposed to the NHS SM [25].

### Step 4: Comparison of the two backward translation versions

Similar to the forward translation committee, a backward translation committee was formed to compare backwards translated versions and resolve discrepancies. Members included a bilingual PhD candidate in the UK who is an evidence implementation trainer in the JBI, a bilingual evidence implementation trainer in the JBI and a Master of Nursing student. The committee also compared the back-translated translation with the original English NHS SM text to assess whether the back-translation correctly reflected the text's original meaning. During the translation process, if the back-translated NHS SM changed the original meaning of the original NHS SM, the relevant back-translated words would again go through steps one to four as described above. This cycle is repeated until the committee members have accepted all backward translations.

### Step 5: Cross-cultural adaptation: expert review and cognitive debriefing interviews

#### Expert review

An expert panel was used to evaluate the conceptual equivalence (clarity) of the introductions, response formats and items of the pre-final Chinese version of the NHS SM. Purposive sampling was used to select bilingual experts who had studied abroad as evidence-based nursing experts.

An invitation and a word document were emailed to the eight experts. The word document consisted of two sections, the first of which was an introduction to the background of the study and a brief description of the NHS SM with links to the original NHS SM for comparison by the experts. The second section evaluates each item's conceptual equivalence and content validity in the Chinese version of the NHS SM. All experts are bilingual and well-versed in evidence-based nursing. The evaluation of conceptual equivalence was dichotomous (explicit or unclear), and all experts whose evaluations were unclear were asked to provide suggestions for changes. If more than 20% of the committee members found it unclear, it was revised and reassessed [26]. To assess the content validity index for each item (I-CVI) and their mean (scale-content validity index/ average [S-CVI/AVE]) of the NHS SM, content validity was evaluated on a four-point scale: 1 = not relevant; 2 = somewhat relevant; 3 = quite relevant; 4 = highly relevant [27, 28]. The I-CVI is calculated by dividing the number of experts giving a score of 3 or 4 (and thus dividing the ordinal scale into relevant and irrelevant) by the total number of experts [29]. There are three ways to calculate S-CVI/Ave, and in this study, we used the approach of summing the I-CVIs and dividing by the number of items and taking the average [29]. A measure has excellent content validity as the minimum of I-CVI exceeds 0.78 [28] with an S-CVI/AVE greater than 0.90 [30].

#### Cognitive debriefing interviews

Linguistic validation assesses how participants understand and respond to the instruments and evaluates the target language version's clarity, comprehensibility, appropriateness, and cultural relevance for the target population [31]. This validation is considered an essential and necessary step before conducting psychometric and statistical tests in local contexts [25, 32].

1. Objectives of cognitive interviewing
  The objective is to examine whether the NHS SM can collect information and identify potential comprehension issues as we intended by probing respondents' understanding and response processes in their responses to the NHS SM.
2. Sample
  Convenience sampling was used. The inclusion criteria for participants were clinical nurses or graduate nursing students who had led an evidence implementation project within the last year. Participants were recruited until data saturation was reached [33]. Six participants took part in the cognitive interviewing process.
3. Cognitive interviewing
  In this study, cognitive interview methods, including observation and verbal probing, were used to complete the language validation [33–35]

The interviews were conducted in Chinese with a postgraduate nursing student (J LAI) who had systematically studied cognitive interviewing following a pre-determined semi-structured interview outline. This semi-structured interview outline was developed after a panel discussion and trial interview with two nursing graduate students who had studied evidence-based nursing.

Before starting the interview, the interviewer briefed the interviewee on the background of the project and the interview process and informed him/her that the process of understanding the model as he/she responded and the entire interview would be recorded. The interviewee signed an informed consent form after expressing their understanding and consent. The interview process was as follows: first, the interviewee read through the NHS model and was asked to think about the project he/she was undertaking. Throughout this process, the interviewer observed the micro-expressions and movements of the interviewee; second, the interviewer used a pre-defined semi-structured interview outline to probe for indication of understanding of the model. (Table 1).

**Table 1:** Cognitive Interview Questions

Details of the cognitive interview questions	
**Overall evaluation of NHS SM**	
1. Which items do you think are easy or difficult to understand?	
2. Do you think any of these items are irrelevant?	
3. Do you think there are any items in these items that make you feel uncomfortable?	
4. Do you feel these questions are out of place in Chinese culture or clinical situations?	
5. Do you have any thoughts or comments on the questionnaire?	
6. What changes would you make to the whole questionnaire?	
**Understanding of NHS SM items**	
1. Can you explain this question in your own words?	
2. What did you think when you answered this question?	
3. Were you confident or unconfident (hesitate) when answering this question?	
4. Which option did you choose and why?	
5. Was the question difficult to understand or easy to understand? Were there any words that were difficult to understand?	
**Comprehension script probe**	
1. How do you understand the term: communication, and can you describe it in your own words or give an example? (Item 2)	
2. How do you understand the term: element? Can you describe it in your own words or give an example? (Item 3)	
3. How do you understand the term: monitoring system? Can you describe it in your own words or give an example? (Item 4)	
4. How do you understand the terms: senior leader and clinical leader? Can you describe them in your own words or give an example? (Item 7 and 8)	
5. How do you understand the term: infrastructure? Can you describe it in your own words or give an example? (Item 10)	
6. How do you understand the term: communication systems? Can you describe them in your own words or give an example? (Item 10)	
7. How do you understand the term: processes? Can you describe them in your own words or give an example? (Item 10)	
**At the End**	
1. Do you have any other comments regarding the questionnaire?	

The cognitive interview used a respondent debriefing technique [33, 35].

Respondents were then asked to recall their project's specifics and assess which factor level they would assign to their project. Respondents initially completed the paper-based sustainability model independently with a pen. The model consists of 10 factors in three dimensions: process, staff, and organisation. Once the model was completed, the interviewee was informed of the overall sustainability score their project had achieved and asked if the score matched what they had in mind for project sustainability (below 55, low likelihood of sustainability, above 55, high likelihood of sustainability, a total model score of 100) and recorded their response. The interviewer then worked through the questions within the sustainability model and the answers provided by the respondent for each item. During this time, the respondent was asked to rate the difficulty of comprehension of each item (1 = easy, 2 = difficult). For difficult items, participants were invited to share any suggestions they thought would improve comprehension of the items. Following this, the interviewer conducted an in-depth interview with the respondent based on the established interview outline.

In order not to influence responses, respondents were only informed of the need to calculate the total NHS SM score to predict the likelihood of sustainability of their project after completing the model. In addition, the semi-structured questions could be flexibly adapted to make the interviews more consistent with the natural order of the conversation.

To ensure the credibility of the interview content, the interviewer used the Vignettes technique of asking respondents to briefly describe their cognitive processes to check whether the condition of the evidence translation project was consistent with the options they had chosen [33]. The interviewer also used the technique of epocher, which is a technique for suspending one's prejudices, making the researcher explore phenomena unbiasedly [36]. Before each interview, the interviewer reminds him/herself to respect the interviewee's point of view and remain neutral. During the interview, the conclusions drawn were fed back to the respondent to ensure that they correctly reflected the respondent's cognitive processes regarding the NHS SM items. The interviews were audio recorded, and the interviewer also made written notes based on their reflections on the interview.

The audio recordings and interview notes were transcribed and collated into an interview file within 24 h by the interviewer and checked again by another author (YN WU) to avoid data being lost in the transcription and collation process. The interviewer used framework analysis [33] to analyse the suggestions and opinions of all respondents on each item and the model and then developed vital questions of understanding and suggestions for revisions. Finally, all questions and suggestions for revision were discussed in a group discussion to discuss whether changes were needed and to arrive at a revision plan.

Based on the expert ratings and cognitive interview results, the research team revised and translated the pre-completed Chinese version of the NHS SM into English. The revised Chinese version of the NHS SM and the English version was translated and sent to the original author of the NHS SM to check whether the Chinese version of the NHS SM was comparable to the original NHS SM in terms of content and measurement intent. The final version of the Chinese version of the NHS SM was developed based on expert ratings, cognitive interviews and comments from the original author of the NHS SM.

## Results

### Results related to the translation (Steps 1–4)

The whole translation process lasted 12 months. In the forward translation, two words were found to be challenging to match in Chinese: "communicated" (item 2 in the model) and “visible” (item 7 in the model), and four words that could be misunderstood in the Chinese healthcare context, which were “system” (item 4 in the model), "senior leadership" (item 7 in the model), "clinical leadership" (item 8 in the model), and "infrastructure" (item 10 in the model). A semantic equivalence issue was identified in the back translation: "any" in the original NHS SM was understood as "all". The forward and translation committees discussed the words: "communicated", "visible", and "any" and agreed that the following changes communicated were changed to "disseminated", "visible", was changed to "obvious", and "all" was changed to "any". The four potentially misunderstood words: "system", "senior leadership", "clinical leadership", and "infrastructure", were tested during the cognitive interviews.

### Results related to Cross-cultural adaptation: expert review and cognitive debriefing interviews (Step 5)

#### Conceptual equivalence and content validity of the Chinese NHS SM

Eight emails were sent for expert review, and all eight experts (Table 2) replied to the emails. The experts evaluated the conceptual equivalence of the instructions, response format, and ten items of the NHS SM, which comprised four options a, b, c, and d. Items one, three, five, and nine were considered unclear by at least 20% (2/8) of the experts, implying a need for revision. For the content validity, the minimum I-CVI exceeded 0.78 (0.88–1), and the AVE was 0.98 with an S-CVI greater than 0.90. (Table 3).

**Table 2:** Demographic characteristics of the expert review

Characteristics	Number
**Sex**	
Female	7
Male	1
**Education**	
Doctoral Degree	3
Master Degree	5
**Position**	
JBI centre directors	2
JBI evidence implementation trainers	2
Head of Faculty Research	2
Head Nurse	2
**Working Years**	
0–10	1
11–20	4
21–30	2
31–40	1

**Table 3:** Date of experts’ ratings and calculation of CVIS

Item	Expert ratings								Number of experts with a rating of 3 or 4	I-CVI
	**A**	**B**	**C**	**D**	**E**	**F**	**G**	**H**		
1	3	4	4	4	4	4	4	4	8	1
2	3	4	4	4	4	4	4	4	8	1
3	4	4	4	4	4	4	3	4	8	1
4	4	4	4	4	4	4	4	4	8	1
5	4	4	3	4	4	4	4	4	8	1
6	4	4	4	4	4	4	4	4	8	1
7	4	4	4	4	4	4	4	4	8	1
8	4	4	4	4	4	4	4	4	8	1
9	4	4	4	4	4	4	4	3	8	1
10	4	4	4	4	4	4	2	4	7	0.88

#### Cognitive interviewing: Testing for the Chinese NHS SM

A round of cognitive interviews was conducted with six people; five nurses and one postgraduate nursing student, all female. The cognitive interviews ranged from 50 min to one hour and 45 min, with an average time of one hour and six minutes. Despite problems with understanding individual items of the model, the model was applied well, and the results of the cognitive interviews are shown below.

## (1) Overall evaluation of the Chinese NHS model

Six respondents completed the model in 10–15 min, with an average of 13 min and 33 s. All indicated that the result of completing the NHS SM sustainability rating aligned with their assessment. Two respondents thought item six: "Staff behaviours toward sustaining the change", where respondents were asked to rate the level at which staff were able to share their ideas, was inappropriate in Chinese culture and clinical settings. One respondent said that nurses in China could express their ideas but were unwilling to do so. Given the heavy clinical workload, putting forward ideas may mean the nurse needs to sacrifice personal time to improve the clinical work. Another respondent stated that employees are not encouraged to express their ideas in the clinical context in China.

## (2) Identified issues of each item of the Chinese NHS model

Of the ten items in the Chinese version of NHS SM, more than half of the respondents found only items 4 and 9 challenging to understand, with the remaining items being found easy to understand by more than half. The terms/points which respondents identified as challenging to understand were: "monitoring system", "small-scale testing", "staff have been involved from the beginning of the change process", and "senior leadership". The results of the cognitive interviewing for each item are shown below.

For item one (Does the change have any other benefits besides helping the patient), one (1/6) respondent had a problem with their understanding of the factor descriptions, which affected their judgement. When recalling the benefits of their project, the respondent did not exclude the description about benefits to the patient.

For item two (Credible benefits of the change), only one (1/6) respondent expressed difficulty understanding it because she was unsure about the difference in the range of detail within the level descriptors. The understanding of the dissemination was probed according to the established script, and no understanding bias occurred.

For item three (Adaptability of improved processes), only one (1/6) respondent expressed difficulty understanding because she felt unsure what the elements in the entry referred to, and further probing by the respondent revealed that the respondent did not have a misunderstanding.

For item four (Is the system for monitoring progress effective), five (5/6) respondents expressed difficulty understanding it because they were unsure what monitoring systems meant. Further probing revealed that some respondents understood it to include some intelligent computer systems, but this did not affect their responses.

For item five (Staff are engaged and trained to sustain change), no interviewees expressed difficulty understanding, but the interviewer detected issues that were not previously anticipated. Some respondents interpreted "the involvement of staff from the beginning of change" as "being implemented from the beginning of the evidence transformation project".

For item six (Staff actions to sustain change), one (1/6) respondent expressed difficulty in understanding it, and a respondent interpreted the “small-scale test” as a “small-scale examinations after training”.

For item seven (Involvement and support of senior leadership), two (2/6) respondents said they had difficulty understanding who the senior leaders were. For item eight (Involvement and support of clinical leadership), no interviewees said they had difficulty understanding. Probing the terms of senior leadership and clinical leadership according to the script probe revealed that two respondents (2/6) had a bias in their understanding of senior leadership, as they perceived both senior and clinical leadership as the department's leader in the hospital.

For item nine (Alignment of change with the strategic objectives and culture of the organisation), three (3/6) respondents indicated it was difficult to understand. Further probing by the interviewer revealed that although they found the term "culture" more challenging to understand, it did not affect their responses. A previously unanticipated understanding issue was identified here, with one interviewee interpreting the "organisation has demonstrated successful sustainability of improvements before", meaning that their change project had been successfully sustained within the organisation.

For item ten (Infrastructure), one (1/6) respondent found it difficult to understand, and the interviewer probed the respondent's understanding of infrastructure and procedures according to the predicted script. Most respondents indicated that their understanding of infrastructure was biased towards hardware facilities. One (1/6) respondent said that in addition to representing the flow, the procedure in Chinese could be understood as software, such as electronic work systems in hospitals. However, these did not influence the respondents' responses.

## (3) Factors influencing the use of the model

The setting of the organisational context influenced the respondents' use of the model. Although all respondents came from a hospital, there was a misunderstanding in the respondents' understanding of the organisation, with half of the respondents understanding it as the ‘hospital’ and half of the respondents understanding it as the ‘department’ in the hospital where their project was implemented.

“For item Nine (Fit with the organisation’s strategic aims and culture), can you describe your understanding of the topic in your words? (Interviewer)

*The changes are the same as some of the goals of improving the quality of care you want to achieve in this area (pause and think) and the cultural climate of this improvement project in your department. (F2)*

I noticed you said department, so how do you understand the word organisation? (Interviewer) It's a small part of the environment where I work. (F2)

Just this tiny part of it, is it? (Interviewer)

*Well, yes. (F2) This is a difficult question for you to understand. Why? (Interviewer) Because it feels like an extensive term, with the strategic objectives of the organisation and the culture of the organisation. This is something that is not mentioned much in China. Well, so it's going to be a conversion. I understand the organisation as the department that I work in. (F2).”*

“You think that item Nine (Fit with the organisation’s strategic aims and culture) is challenging to understand. Why? (Interviewer)

*It's the word of organisation; it's the idea that it can have more than one meaning. I think it's important to clarify this; otherwise, I don't know what this organisation exactly means. (F3)*

So, what did you think about this entry when you answered it? (Interviewer)

*When I started looking at the item, I wondered whether it was a department or a hospital. Because the head doctor and head nurse of different departments think differently, some department heads, for example, don't think evidence-based practice is useful, but some department heads will think it's useful. So, you're still supporting it in terms of the wider hospital environment. (F3)*

So, you're just defining it as a hospital. (Interviewer) Yes, it is. (F3)”

Differences in specific scenario settings will further influence their responses. This is because even within the same hospital, different department heads may have different styles, which can influence respondents' perceptions when implementing their projects and then affect their judgement when evaluating NHS SM.

“In fact, according to the title and options of this item, I feel that it mainly wants to express whether there are channels for employees to share their ideas in the process of achieving this change and then whether they can be recognised for sharing them and whether they will be empowered to implement them. First of all, no matter the hospital construction or discipline development, the leadership, such as the director of the nursing department and the head nurse, are very encouraging for people to conduct evidence implementation projects. Hence, staff might be allowed to share, but there is a situation where no one will share their opinion since the staff do not want to do these things because of the heavy workload. You might need to spend extra time in China to do these things (EBP). So, the staff just do not want to share their opinion about it even if their ideas will be accepted and can be implemented in the clinic. However, the item does not have this option. (F1)”

“For item six, in the department where I work, everyone is just doing these things (EBP) more passively. ah… (F4)

Is it relative to the general environment in China? (Interviewer)

The whole environment, the encouragement of expression (thinking). Just the expression of this question is not in line with the clinical scenario in China, that is, in the general environment. I do not think Chinese (leaders) are too keen on (staff) expressing themselves, well, encouraging (staff) to express this thing. I think, um, it does not quite fit (the Chinese clinical scenario). (F4).”

Based on the results of the expert review and cognitive interviews, items one, three, four, five, seven, nine, and ten (items 1,3, 4, 5, 7, 9 and 10) were revised through group discussions, and the original NHS SM developers examined the final Chinese version of the revision. The seven items changed in the process are shown in Table 4.

**Table 4:** Modified items after experts review and cognitive interviewing

Source item	Chinese items before experts review and cognitive interviewing	Revised items	Reasons for revision
1 Benefits beyond helping patients d The benefits that we have identified are only directly related to helping patients. We have not identified any other benefits that this initiative could bring	1 **该变革除了帮助患者以外, 是否有其他益处** d **我们还未明确变革能够带来的其他益处。**	**1 该变革除了帮助患者以外的益处** d **除了帮助患者以外**, 我们还未明确该变革能够带来的其他益处。	During the evaluation process by the back translation expert committee, the translation team felt that it was possible that the entry already conveyed that the benefits did not include helping patients and avoiding overloading the interviewees with text; option d: only benefits that could identify helping patients were removed However, one respondent in the cognitive interview included the benefit of helping patients when judging the benefit of the change because option d did not mention the changed benefit of helping patients. After discussion by the translation team, it was decided to leave the original scale formulation unchanged to allow respondents to exclude the benefit of helping patients when assessing the number of benefits of the items
	1 Does the change have any other benefits besides helping the patient? d **We have not identified any other benefits that this initiative could bring**	1 Does the change have any benefits beyond helping patients? d **We can only be sure that the benefits of the change will help patients**, and no other benefits have been identified	
3 Adaptability of improved process a The improved process can adapt to link in with and even support other organizational changes. It would not be disrupted if specific individuals or groups left the project. Its focus will continue to meet the improvement needs of our organisation	3 改进流程的适应性 a 改进的流程能适应组织环境, 甚至支持**其他组织变革**。如果特定的人或小组离开项目, 它不会被中断。它的重心将会继续满足我们组织的改进需求。	3 改进流程的适应性 a 改进的流程能适应**组织的其他变化**, 甚至支持它, 如果特定的人或小组离开该变革项目, 流程不会被中断。流程的重心将会不断地满足我们组织的改进需求。	change can be translated as “变革” and “变化”. “变革” in Chinese tends to be understood as organisational innovation, such as a quality improvement project. "变化” in Chinese refers to "any change in the organisation", such as organisational structure and staff changes. After communicating the issue to the original authors of the NHS SM, we decided to translate the "change" into "变化” Moreover, It has been pointed out that "other organisational changes” can not only refer to “other changes in the organisation”(组织的其他变革) but can also be understood as "changes in another organisation” (其他组织的变革). For further clarification, we decided to translate “other organisational change” (其他组织变革) as “other changes in the organisation” (组织的其他变化)
	3.Adaptability of improved processes a. Improved processes can adapt to the organisational environment and **even facilitate other changes**. If a specific individual or group leaves the project, the project will not be interrupted. The focus of this work remains on meeting the organisation's improvement needs	3 Adaptability of improved processes a. mproved processes can be adapted to **the organisational changes and even facilitate them**. If a particular individual or group leaves the project, the project will not be interrupted. The focus of this work will continue to meet our organisational needs for improvement	
4 Effectiveness of the system to monitor progress a There is a system in place to provide evidence of impact, including benefits analysis, monitor progress and communicate the results. This is set up to continue beyond the formal life of the project	**4 监测进展的系统是否有效** a有一个能反馈**变革所带来影响的系统**, 包括效益分析、监测进展并传播结果。该系统是为了项目在正式结束后能够继续实施而设置的。	**4 监测体系的有效性** a 有一个能反馈**变革影响的体系**, 它包括效益分析、监测进展和传播结果。该体系是为了项目在正式结束后能够继续实施而建立的。	One respondent indicated that “monitoring systems” (监测系统) can also be understood in Chinese as “electronic monitoring applications”, although this understanding did not affect the respondent's answer model. However, for further clarification, we decided to replace “监测系统” with “监测体系”, although they can both be translated as monitoring systems in English, "体系**”** in Chinese does not include intelligent electronic monitoring applications but refers to different things linked together to form a whole Given that the options in item 4 and the description of the question have a role in explaining this monitoring system, such as "monitoring progress", we have removed "progress** (进展**)” from the title of item 4
	**4 Is the system for monitoring progress effective?** a there is a system for feedback on the impact of change, including benefit analysis, monitoring progress and disseminating results. The system is set up so that the project can continue after its official conclusion	**4 Effectiveness of the monitoring system** a There is a system for feedback on the impact of change, including benefit analysis, monitoring progress and dissemination of results. The system is designed to enable the project to continue after its formal closure	
5 Staff involvement and training to sustain the process a Staff have been involved from the beginning of the change process. They have helped to identify any skill gaps and have been able to access training and development so that they are confident and competent in the new way of working	**5 员工是否参与并接受了培训以维持变革** a 员工**从变革开始**就参与其中, 他们帮助识别所有的技能差距, 并且能够获得培训和成长, 因此他们充满信心并能够胜任新的工作方式。	**5 员工参与和接受了培训以维持变革** a 员工**从变革的初始阶段**就参与其中。他们帮助识别任何技能差距, 并且能够获得培训和成长, 因此他们对新的工作方式充满信心并能够胜任它。	Some respondents interpreted "being involved from the beginning of change**” (**从变革一开始) as being involved at the beginning of the implementation of change, excluding the innovation and design phases of change. Therefore, we decided to replace “being involved from the start of the change”(从变革一开始) with “the initial stages of the change” (从变革的初始阶段).“从变革的初始阶段” in Chinese there is more emphasis on involvement from the beginning of the project rather than during the implementation phase of the project
	5 Staff are engaged and trained to sustain change a Employees are **involved from the beginning of the change**, they help identify all skills gaps, and are able to train and grow so they feel confident and competent in the new way of working	5 Staff are engaged and trained to sustain change a Staff are **involved from the initial stages of the change.** They help identify any skills gaps and have access to training and growth so they are confident in the new way of working and competent to do it	
7 Senior leadership engagement and support	**7 高级领导**的参与和支持Involvement and support of **senior leadership**	**7 组织高层领导**的参与和支持Involvement and support of **senior leadership of the organisation**	Two respondents equated senior leadership with clinical leadership. For further clarification, we decided to replace senior leadership **(**高级领导)with senior leadership of the organisation (组织高层领导). 高级领导 and 高层领导 mean the same thing in Chinese, but senior leader is used more frequently in Chinese. Adding organisation** (组织)** can emphasise that it is the leader of the whole work unit rather than the department
9 Fit with the organisation’s strategic aims and culture a The goals of the change are clear and have been shared widely. They are consistent with and support the organisation’s strategic aims for improvement. The organisation has demonstrated successful sustainability of improvements before and has a ‘can do’ culture	**9变革是否与组织的战略目标和文化相符** a 变革的目标明确, 并得到了广泛的认同。它们与该组织改进的战略目标一致并能够支持该战略目标。该组织之前已成功证明了**改进**的可持续性, 并且有一种 “能做”的文化	**9与组织的战略目标和文化相符** a变革的目标明确, 并得到了普遍认同。它们与组织为了改进的战略目标一致并能够支持该目标。该组织之前有成功维持过**变革**, 并且有一种 “能做”的文化。	One respondent interpreted improvement (改进)as an improvement project undertaken by the project leader. For further clarification, we reviewed improvement (改进) to change (变革), emphasising that the organisation has a history of successfully sustaining change rather than improvement projects undertaken by project leaders that have previously been successfully sustaining the organisation
	9. Alignment of change with the strategic objectives and culture of the organization a The goal of the change is clear and widely recognised. They align with and support the organisation's improved strategic objectives. The organisation has successfully demonstrated the sustainability of **improvements** before and has a "can-do" culture	9. Alignment with the strategic objectives and culture of the organization a The goal of the change is clear and widely recognised. They are aligned with and can support the organisation's strategic goal of improvement. The organisation has had previous success in sustaining **change** and has a "can do" culture	
10 Infrastructure a Staff are confident and trained in the new way of working. Job descriptions, policies and procedures reflect the new process and communication systems are in place. Facilities and equipment are all appropriate to sustain the new process	**10 基础设施** a 员工对新的工作方式充满信心, 并接受了培训。工作说明、政策和**程序**能够体现出新的工作流程, 且沟通系统已就位。设施和设备都适合维持新的工作**流程**。	**10 基础建设** a 员工对新的工作方式充满信心并且接受了培训。工作说明、政策和**流程**能够体现新的工作方式, 且沟通系统已就位; 设施和设备都适合维持**新的变革**。	Most respondents tended to understand “infrastructure” (基础设施) as a hardware facility, although the descriptions in the options could help them understand it as both soft and hard facilities. And one respondent indicated that infrastructure (基础建设) was more easily understood; therefore, for further clarification, we decided to use "infrastructure" (基础建设) instead of “infrastructure” (基础设施) Similar to the system, some respondents thought that the "procedure" (程序) could also be understood as an electronic application in Chinese, and for further clarification, we decided to modify “procedure” (程序) to “processes” (流程). To avoid duplication and misunderstanding of "processes" (流程) and “new processes" (流程). We have translated “process” into “change” (变革) based on the fact that the “new process” in NHS SM means “change”
	**10 Infrastructure** a Staff are confident and trained in new ways of working. Job descriptions, policies and **procedures** that reflect new workflows and communication systems are in place. Facilities and equipment are suitable for maintaining **new processes**	**10 Infrastructure** a Staff are confident and trained in new ways of working. Job descriptions, policies and **processes** that reflect new workflows and communication systems are in place. Facilities and equipment are suitable for maintaining **new change**	

## Discussion

There has been an increase in the number of EBPs in China in recent years, but few of these EBPs mention the sustainability of the project [20], and there is no tool to evaluate the sustainability of EBPs or continuous quality improvement projects in China [18].

As such, this study describes the process of translation and cross-cultural adaptation of the NHS SM for use in China by following rigorous translation and linguistic validation guidelines, including cognitive interviews [25, 33]. The main objectives of this study were to develop a Chinese version of the NHS SM and to validate the NHS SM's conceptual equivalence and linguistic validity in a Chinese healthcare scenario.

Meetings with the translation committee on the forward and reverse translation process helped to identify any differences and improve the quality of the translation [25]. Although most of the NHS SM items were relatively easy to translate in forwarding translation, the committee found some differences between the first and second forward translations. After refining each stage of the translation process, the committee identified some inconsistencies between the English and Chinese versions. All discrepancies were resolved by group discussions until every member of the group reached a consensus. For example, the communication (沟通) terminology in the original NHS SM item two was challenging to match in Chinese in that context because, according to the Xinhua dictionary, "communicate" in Chinese means to make both parties understand each other, such as ideas, culture [37], yet “communication” in NHS SM means much more than just two parties. It took four rounds of group discussion and confirmation with the author of NHS SM before we finally settled on translating it into “dissemination” (传播), which is the process of transmitting information, intelligence, opinions, feelings and directly or indirectly between people or groups of people with the help of verbal and non-verbal symbols in Chinese [38].

Content equivalence between the pre-final translation and the original version can be further improved through expert review, and the composition of the expert committee needs to be carefully considered because it is essential for achieving cross-cultural equivalence, such as the expert's knowledge of the study area and the expert's familiarity with the target subjects of the study [25]. This criterion was met in this study by inviting eight Chinese EBP experts who had the experience of studying abroad and proficiency in English to assess the equivalence and content validity of the pre-translated NHS SM. The experts rated the pre-translated NHS SM as good overall but identified minor issues, such as the ordering of statements and the use of pronouns, which were revised by the translation team.

The primary purpose of the cognitive interview was to test whether the developed Chinese version of the NHS SM was understood following our measurement intentions by understanding the respondents' cognitive processes towards the NHS SM. Some predicted questions were given more detailed information through the cognitive interviews, while some unpredicted understanding issues were also identified. These issues were addressed through group discussion, interviewee, and expert suggestions, and the content equivalence between the Chinese and the original versions was verified together with the NHS SM. This study used a cognitive debriefing technique to conduct the cognitive interview [35, 39], which enabled some quantitative data to be collected before the formal interviews began. For example, how long it took respondents to complete the model, how well the total item persistence scores matched their ratings, and which items were difficult or easy to understand. These quantitative results can be used not only as a basis to help interviewers conduct interviews but also as an aid in determining whether the NHS SM can initially test the sustainability of the evidence translation project and whether the respondents easily understand it.

Of six respondents from the same hospital, half understood the ‘organisation’ as ‘department’ and half as ‘hospital’. This difference may be related to how EBP is implemented and the clinical culture in China. Currently, in China, EBPs are primarily carried out on a departmental basis rather than throughout the hospital. Therefore, the smooth implementation and maintenance of EBP mainly depend on departmental leaders' support [40]. In addition, due to the traditional Chinese culture, Chinese clinical departmental leaders present a centralised form of paternalism, which means that if the head nurse does not agree to conduct or continue to maintain the implementation of EBP, then EBP will not be introduced [41].

The level of understanding of the organisation influenced the respondent's answers to the models’ items. Two respondents in the cognitive interview felt that the description of NHS SM item six on whether employees could express their ideas and opinions needed to be more consistent with Chinese culture in healthcare. However, it is worth noting that there are also differences between the two interviewees' statements. One interviewee felt that the hospital and department heads allowed and encouraged staff to express their ideas, but that staff were reluctant to do so because they were already busy with their nursing routines, and giving their opinions would increase their workload and sacrifice their own personal time to do so. Another interviewee stated that staff in his department are only passive in implementing EBP and felt that, generally speaking, Chinese leaders need to encourage staff to express themselves. This difference may be related to the leadership style of the respondent's supervisors [42]. However, the management style of clinical scenarios in China is dominated by the traditional type of staff obedience [43, 44]. Some senior leaders are transitioning to transformational leadership by introducing the concept of transformational leadership [45, 46].

Given that the NHS SM measures the sustainability of the evidence translation project itself, the measurement of entry six needs to be more integrated with the leadership style of the specific scenario in which the project is implemented rather than judging the sustainability of the evidence translation project from the perspective of the leadership style of China as a whole. As such, the translation team did not make changes to item six.

### Implications on nursing practice

The NHS SM is easy to use and can facilitate and support evidence-based practice implementation and maintenance. It helps project managers better understand project implementation's strengths and weaknesses by scoring the ten key factors that affect project sustainability [24]. For example, the user can identify the organisation's culture and prevailing leadership style based on the model. If the organisation's culture and leadership style undermine the project's sustainability, the user can take further action based on the specific description of the items and their options to create an organisational scenario that encourages EBP implementation and maintenance [47].

### Limitations

Although our study is robust, it has limitations. First, the cognitive interviews were conducted in a tertiary hospital in Guangzhou, a JBI-endorsed healthcare organisation with more training and opportunities for EBP projects than hospitals in other smaller cities in China. Therefore, participants in this study may have been more exposed to the knowledge of conducting EBP than other samples. Second, the NHS SM is a diagnostic model developed for the sustainability of EBP or quality improvement projects. We have translated it and made it as easy to understand as possible. However, there is no denying that the model requires a high level of knowledge of evidence-based healthcare from its users.

## Conclusion

In this study, the English version of the NHS SM was translated into Chinese, and language validation was completed in a Chinese healthcare setting according to rigorous and systematic guidelines. The reliability of the translation and linguistic validation was reinforced by the involvement of the original tool developers in the process [25]. This study provides insight into the use of the NHS SM in the local Chinese healthcare setting and highlights the importance of understanding the different contexts in which the tool was developed and used. Large-scale testing is needed further to evaluate the Chinese version of the NHS SM.

## Supplementary Information

**Additional file 1: Supplementary 1.** The Chinese version of the NHS Sustainability Model (English).

**Additional file 2: Supplementary 2.** The Chinese version of the NHS Sustainability Model.

## Data Availability

The datasets used and analysed the current study are available from the corresponding author upon reasonable request.

## Abbreviations

EBP: Evidence-based practice
NHS: National Health Service
SM: Sustainability Model
JBI: Joanna Briggs Institute

## References

[1] Grol R, Grimshaw J (2003). From best evidence to best practice: effective implementation of change in patients' care. Lancet (London, England).

[2] Lennox L, Maher L, Reed J (2018). Navigating the sustainability landscape: a systematic review of sustainability approaches in healthcare. Implement Sci.

[3] Cheng L, Feng S, Hu Y (2017). Evidence-based nursing implementation in Mainland China: a scoping review. Nurs Outlook.

[4] Zhang L, Zhou C, He L, Wu Y, Xie G, Chen P (2021). Implementation of strategies to improve nutritional intervention for patients with cancer treatment-related oral mucositis: a best practice implementation project. JBI Evid Implement.

[5] Wu Y, Li W, Stephenson M, Cong W, Zhou C (2020). Pre-treatment assessment for patients with breast cancer undergoing chemotherapy: a best practice implementation project. JBI Evid Synth.

[6] Yang SH, Mu PF, Wu HL, Curia M (2019). Fluid balance monitoring in congestive heart failure patients in hospital: a best practice implementation project. JBI Database Syst Rev Implement Rep.

[7] Ammerman A, Smith TW, Calancie L (2014). Practice-based evidence in public health: improving reach, relevance, and results. Annu Rev Public Health.

[8] Ham C, Kipping R, McLeod H (2003). Redesigning work processes in health care: lessons from the National Health Service. Milbank Q.

[9] WiltseyStirman S, Kimberly J, Cook N, Calloway A, Castro F, Charns M (2012). The sustainability of new programs and innovations: a review of the empirical literature and recommendations for future research. Implement Sci : IS.

[10] Ament SM, de Groot JJ, Maessen JM, Dirksen CD, van der Weijden T, Kleijnen J (2015). Sustainability of professionals' adherence to clinical practice guidelines in medical care: a systematic review. BMJ Open.

[11] Shelton RC, Cooper BR, Stirman SW (2018). The Sustainability of evidence-based interventions and practices in public health and health care. Annu Rev Public Health.

[12] Chambers DA, Glasgow RE, Stange KC (2013). The dynamic sustainability framework: addressing the paradox of sustainment amid ongoing change. Implement Sci.

[13] Scheirer MA (2013). Linking sustainability research to intervention types. Am J Public Health.

[14] Proctor E, Luke D, Calhoun A, McMillen C, Brownson R, McCrary S (2015). Sustainability of evidence-based healthcare: research agenda, methodological advances, and infrastructure support. Implement Sci.

[15] Birken SA, Haines ER, Hwang S, Chambers DA, Bunger AC, Nilsen P (2020). Advancing understanding and identifying strategies for sustaining evidence-based practices: a review of reviews. Implement Sci.

[16] Greenhalgh T, Robert G, Bate P, Kyriakidou O, Macfarlane F. How to spread good ideas: a systematic review of the literature on diffusion, dissemination and sustainability of innovations in health service delivery. London: NHS SDO Programme; 2004.

[17] Scheirer MA, Dearing JW (2011). An agenda for research on the sustainability of public health programs. Am J Public Health.

[18] DENG BO, LIU Ning, Linb Y (2021). Scoping review of clinical practice project sustainability assessment tools (In Chinese). J Nurs (China).

[19] Lai J, Brettle A, Zhang Y, Zhou C, Li C, Fu J (2022). Barriers to implementing evidence-based nursing practice from the hospitals' point of view in China: A regional cross-sectional study. Nurse Educ Today.

[20] Zhou Yingfeng, Huang Na, Hu Yan, Xing Weijie, Zheng Z (2020). Studies on evidence implementation in clinical nursing practice in China: a scoping review in Chinese. Chin Nurs Manage.

[21] Lennox L, Linwood-Amor A, Maher L, Reed J (2020). Making change last? Exploring the value of sustainability approaches in healthcare: a scoping review. Health Res Policy Syst.

[22] Maher L, Gustafson D, A. E. Sustainability Model and Guide. Coventry: NHS Institute for Innovation and Improvement; 2010.

[23] Doyle C, Howe C, Woodcock T, Myron R, Phekoo K, McNicholas C (2013). Making change last: applying the NHS institute for innovation and improvement sustainability model to healthcare improvement. Implement Sci.

[24] Silver SA, McQuillan R, Harel Z, Weizman AV, Thomas A, Nesrallah G (2016). How to sustain change and support continuous quality improvement. Clin J Am Soc Nephrol.

[25] Sousa VD, Rojjanasrirat W (2011). Translation, adaptation and validation of instruments or scales for use in cross-cultural health care research: a clear and user-friendly guideline. J Eval Clin Pract.

[26] Topf M (1986). Three estimates of interrater reliability for nominal data. Nurs Res.

[27] Lenz ER (2010). Measurement in nursing and health research.

[28] Lynn MR. Determination and quantification of content validity. Nur Res. 1986;35(6):382–6. 3640358

[29] Polit DF, Beck CT (2006). The content validity index: are you sure you know what's being reported? Critique and recommendations. Res Nurs Health.

[30] Waltz CF (2005). Measurement in Nursing and Health Research.

[31] Acquadro C, Conway K, Giroudet C, Mear I. Linguistic validation manual for health outcome assessments. France: Mapi Institute; 2012.

[32] Egger-Rainer A (2019). Enhancing validity through cognitive interviewing. A methodological example using the epilepsy monitoring unit comfort questionnaire. J Adv Nurs.

[33] Collins D (2015). Cognitive interviewing practice.

[34] Nichols E, Hunter Childs JJFM (2009). Respondent debriefings conducted by experts: a technique for questionnaire evaluation. Field Methods.

[35] Willis GB (2004). Cognitive interviewing: Thousand Oaks.

[36] Fernandez AV (2020). Embodiment and objectification in illness and health care: taking phenomenology from theory to practice. J Clin Nurs.

[37] Chinese academy of social sciences and languages institute. Xinhua Dictionary 12th Edition. Beijing: The Commercial Press; 2020.

[38] Jang I-S, Park M (2016). Knowledge management, beliefs, and competence on evidence-based practice, evidence-based decision making of nurses in general hospitals. Korean J Adult Nurs.

[39] Liu Y, Hinds PS, Wang J, Correia H, Du S, Ding J (2013). Translation and linguistic validation of the pediatric patient-reported outcomes measurement information system measures into simplified Chinese using cognitive interviewing methodology. Cancer Nurs.

[40] Zhao J, Bai W, Zhang Q, Su Y, Wang J, Du X (2022). Evidence-based practice implementation in healthcare in China: a living scoping review. Lancet Reg Health West Pac.

[41] Fu Y, Wang C, Hu Y, Muir-Cochrane E (2020). The barriers to evidence-based nursing implementation in mainland China: A qualitative content analysis. Nurs Health Sci.

[42] Aarons GA, Green AE, Trott E, Willging CE, Torres EM, Ehrhart MG (2016). The roles of system and organizational leadership in system-wide evidence-based intervention sustainment: a mixed-method study. Adm Policy Ment Health.

[43] Farh J-L, Cheng B-S. A Cultural Analysis of Paternalistic Leadership in Chinese Organizations. In: Li JT, Tsui AS, Weldon E, editors. Management and Organizations in the Chinese Context. London: Palgrave Macmillan UK; 2000. p. 84–127.

[44] Chan ZC (2007). Are you ready to be a nursing leader?. J Nurs Manag.

[45] Xu X, Zhang Y, Zhou P, Zhou X (2022). Developing a leadership and management competency framework for nurse champion: a qualitative study from Shanghai China. J Nurs Manage.

[46] Zhang F, Peng X, Huang L, Liu Y, Xu J, He J (2022). A caring leadership model in nursing: a grounded theory approach. J Nurs Manag.

[47] Higuchi KS, Davies B, Ploeg J (2017). Sustaining guideline implementation: a multisite perspective on activities, challenges and supports. J Clin Nurs.

