# Supplementary material for: Translation and cross-cultural adaptation of the National Health Service Sustainability Model to the Chinese healthcare context
Source: BMC Nurs. 2023 Apr 15;22:124. doi: 10.1186/s12912-023-01293-x (PMC10105950; doi:10.1186/s12912-023-01293-x)
Supplement: Supplementary file 1 — Additional file 1: Supplementary 1. The Chinese version of the NHS Sustainability Model (English). [file 12912_2023_1293_MOESM1_ESM.docx]

**Copyright statement.**

**The Chinese version of the NHS Sustainability Model (in English) is copyright of Jie Lai and the Southern Collaborating Centre for Evidence-Based Care. To use it, please contact the corresponding author; for the original version of the NHS Sustainability Model, please contact Professor Maher.**

**NHS sustainability model**

**I. Process**

**1. P1: Does the change have any benefits beyond helping patients**

Question Description:

- What are the benefits of the change beyond helping patients?
- For example, do these changes reduce waste and avoid duplication?
- Does it make things smoother?
- Will employees notice the changes in their routine?

1. In addition to helping patients, we can clearly say that the change has many benefits, such as reducing waste, increasing efficiency, or making work easier.
2. We can clearly say that the change has some benefits in addition to helping patients. Less waste and easier work, for example, but the benefits are not great.
3. We can clearly say that the change has one or two benefits in addition to helping patients.
4. We can only be sure that the benefits of the change will help patients, and no other benefits have been identified.

**2. Credible benefits of the change**

- Are these benefits visible to patients, employees, and organizations?
- Do employees believe in the benefits?
- Can all employees clearly describe all the benefits?
- Is there evidence that such changes have been made elsewhere?

1. The benefits of change not only are supported by evidence and recognized by stakeholders but also widely disseminated, rapid and visible. Employees were able to fully describe the expected benefits of this initiative.
2. The benefits of change are not widely disseminated or immediately apparent, even when they are supported by evidence and recognized by stakeholders.
3. The benefits of change are not widely disseminated or immediately apparent, and even when they are supported by evidence, they are not widely recognized by stakeholders.
4. The benefits of change are not widely disseminated or immediately apparent, and they are neither supported by evidence nor widely recognized by stakeholders.

**3. Adaptability of improved processes**

- Can the new process overcome or be disrupted by internal pressures within the organization?
- Will change continue to be effective in meeting continuing needs?
- Does change require specific individuals or groups, technologies, funding, etc, to keep it going.
- When these are removed, can change continue?

1. Improved processes can be adapted to the organizational changes and even facilitate them. If a particular individual or group leaves the project, the project will not be interrupted. The focus of this work will continue to meet our organizational needs for improvement.
2. Improved processes can be adapted to the organizational environment and even facilitate other changes, but if a particular individual or group leaves the project, the project will be disrupted. The element of this work will continue to meet our organizational needs for improvement.
3. New processes are difficult to adapt to other organizational changes. If a particular individual or group leaves the project, the project will be interrupted.
4. New processes cannot adapt to other organizational changes. If a particular individual or group leaves the project, the project will be interrupted.

**4.** **Effectiveness of the monitoring system**

- Does change require special monitoring systems to identify and continuously monitor progress?
- Is there a feedback system to enhance benefits and progress and initiate new or further actions?
- Is there a system in place to continue monitoring progress after the formal closure of the project?
- Do the results of the changes spread to patients, employees, organizations, and the broader healthcare community?

1. Have a system for feedback on the impact of change, including benefit analysis, monitoring progress and dissemination of results. The system is designed to enable the project to continue after its formal closure.
2. There is a system for feedback on the impact of change, including benefit analysis, monitoring progress and dissemination of results. The system is not designed to allow the project to continue beyond its official closure.
3. There is a system for feedback on the impact of change and for monitoring progress, however, this information is disseminated only within the core project team. The system is not designed to allow the project to continue beyond its official closure.
4. There is only one very incomplete system to monitor progress, and that system is terminated with the project. There is no system to disseminate results.

**II. STAFF**

**5 Participation and training of staff in the project**

- Do employees play a role in innovation, design and implementation of change?
- Have employee ideas been taken into account from the start of the change?
- Is there a training and development infrastructure to identify gaps in skills and knowledge, and are staff receiving such education and training to drive change?

1. Staff have been involved from the start of change, helping to identify all skills gaps and have access to training and growth, so they are confident and competent in new ways of working.
2. Staff have been involved since the beginning of the change and have helped to identify skills gaps, but they have not been trained or developed in new ways of working.
3. Staff have not been involved since the beginning of the change, but they have been trained in new ways of working.
4. Staff have not been involved since the beginning of the change, have not been trained in new ways of working and have not grown in new ways of working.

**6 Staff actions to sustain change**

- Throughout the change process, are employees encouraged to express themselves, are they able to do so regularly, and are their opinions being taken up?
- Do employees see change as a better way to work that they want to keep in the future?
- Have employees been trained and authorized to conduct small-scale tests (PDSA) based on their ideas to see if there are other areas for improvement?

1. Employees can regularly share their ideas, some of which have already been incorporated into the project. They think change is a better way to work, and they have been mandated to conduct small-scale testing (planning, implementation, research, action).
2. Employees can regularly share their ideas, some of which have already been incorporated into the project. They think change is a better way to work, but they are not authorized to conduct small-scale testing (planning, implementation, research, action).
3. Staff were able to share their ideas on a regular basis, but their ideas were not taken up during the project. They don't think change is a better way to work, and they're not authorized to conduct small-scale testing (planning, implementing, research, action).
4. Staff do not think they can share ideas. They don't think change is a better way to work, let alone mandate small-scale testing (planning, implementation, research, action).

**7 Involvement and support of senior leadership of the organization**

- Are senior leaders trustworthy, influential, respected and trustworthy?
- Are they involved in this initiative and do they understand and promote it?
- Are they respected by their peers and can they influence others to join?
- Do they take personal responsibility for helping to overcome obstacles and do they take the time to help ensure the success of change?

1. Organizational leadership is highly engaged in and very supportive of the change process. They used their influence to spread the benefits of change and overcome any obstacles. Employees regularly share information with their leaders and actively seek leadership advice.
2. Organizational leadership is highly engaged in and very supportive of the change process. They used their influence to spread the benefits of change and overcome any obstacles. But employees often don't share information or seek advice from their leaders.
3. Organizational leaders are involved to some extent in the change process, but their support for change is not significant. They use their influence to spread the benefits of change, but they cannot be relied upon to overcome any obstacle if things become difficult. Employees often don't share information or seek advice from their leaders.
4. Organizational leaders are not involved or see their support for the change process. They have not used their influence to spread the benefits of change or overcome any of the obstacles. Employees often don't share information or seek advice from their leaders.

**8. Involvement and support of clinical managers**

- Are clinical managers trustworthy, influential, respected and trustworthy?
- Are they involved in this initiative and do they understand and promote it?
- Are they respected by their peers and can they influence others to join?
- Do they take personal responsibility for helping to overcome obstacles and do they take the time to help ensure the success of change?

1. Clinical manager is highly involved and supportive of the change process. They use their influence to spread the benefits of change and overcome any obstacles. Staff regularly share information with clinical managers and actively seek advice.
2. Clinical managers are highly involved and supportive of the change process. They use their influence to spread the benefits of change and overcome any obstacles. But staff often do not share information with clinical managers or seek their advice.
3. Clinical managers are involved to some extent in the change process, but their support for change is not significant. They use their influence to spread the benefits of change, but they cannot be relied upon to overcome any obstacle if things become difficult. Staff often do not share information with clinical managers or seek advice.
4. Clinical managers are not involved or see their support for the change process. They have not used their influence to spread the benefits of change or overcome any of the obstacles. Staff often do not share information with clinical managers or seek advice.

**III. ORGANIZATION**

**9. Alignment of change with the strategic objectives and culture of the organisation**

- Are the goals of change clear and recognized?
- Do they contribute significantly to the overall strategic objectives of the organization?
- Is improvement important to the organization and its leadership?
- Has the organization been successful in sustaining improvements before?

1. Change is well targeted and widely accepted. They are consistent with and able to support the improved strategic objectives of the organization. The organization has previously successfully demonstrated the sustainability of improvements and has a "can do" culture.
2. The goals of change are clear and widely shared. They are consistent with and able to support the improved strategic objectives of the organization. The organization has yet to succeed in sustaining previous improvements and has no "can do" culture.
3. The goals of change are clear and widely shared. They are not aligned with the organization’s strategy, so we do not know if the goal of change supports any of the organization’s goals for improvement The organization has yet to succeed in sustaining previous improvements and has no "can do" culture.
4. The objectives of change are unclear and not widely shared. They are not aligned with the organization’s strategy, so we do not know if the goal of change supports any of the organization’s goals for improvement. The organization has yet to succeed in sustaining previous improvements and has no "can do" culture.

**10 Infrastructure**

- Are employees adequately trained and able to work in new ways?
- Are there sufficient facilities and equipment to support the new procedures?
- Are there new requirements in the job description?
- Are there policies and procedures in place to support the new working methods?
- Is there an appropriate dissemination system?

1. Staff are confident and trained in new ways of working. Job descriptions, policies and procedures that reflect new workflows and communication systems are in place. Facilities and equipment are suitable for maintaining new workflows.
2. Staff are confident and trained in new ways of working. However, the job descriptions, policies and procedures did not reflect the new workflow. Some communication systems are in place. Facilities and equipment are suitable for maintaining new workflows.
3. Staff are confident and trained in new ways of working. However, the job descriptions, policies and procedures did not reflect the new workflow and did not adequately support its communications system. Facilities and equipment are not suitable for maintaining new workflows.
4. Staff have not been trained in new workflows and have no confidence in new ways of working. Job descriptions, policies and procedures did not reflect the new workflow and did not adequately support its communications system. Facilities and equipment are not suitable for maintaining new workflows.
