# Supplementary material for: Translation and cross-cultural adaptation of the National Health Service Sustainability Model to the Chinese healthcare context
Source: BMC Nurs. 2023 Apr 15;22:124. doi: 10.1186/s12912-023-01293-x (PMC10105950; doi:10.1186/s12912-023-01293-x)
Supplement: Supplementary file 2 — Additional file 2: Supplementary 2. The Chinese version of the NHS Sustainability Model. [file 12912_2023_1293_MOESM2_ESM.docx]

**版权声明：**

**NHS可持续模型的中文版的版权归南方医院南方循证护理中心及赖杰所有。如需使用，请联系通讯作者获取授权；如需使用NHS可持续性模型的原始英文版本，请与Maher教授联系。**

**请您在仔细阅读每个问题及其描述后，在a、b、c、d四个选项中选择一个最接近您的项目实际情况的选项，并在其左侧打勾☑。**

**一、过程**

**1该变革除了帮助患者以外的益处**

问题描述

·该变革除了帮助患者以外，还有其他益处吗？

·比如，这些变革是否减少了浪费和避免了重复？

·它有没有让工作变得更顺畅？

·员工会留意到他们日常工作中的变化吗？

- a除了帮助患者以外，我们能明确说出该变革还有诸多益处，比如减少浪费，提高效率或者让工作变得更容易。
- b除了帮助患者以外，我们能明确说出该变革还有一些益处。比如减少浪费和使工作变得更容易，但益处并不多。
- c 除了帮助患者以外，我们能明确说出该变革还有一、两个益处。
- d 除了帮助患者以外，我们还未明确该变革能够带来的其他益处。

**2益处的可信度**

问题描述

·这些益处对患者、员工和组织来说，是显而易见的吗？

·员工相信这些益处吗？

·所有员工都能清晰地描述该变革带来的一系列益处吗？

·有证据表明已经在其他地方实现了这类型的变革吗？

- a变革的益处不仅有证据支持和被利益相关者认可，而且能立即显现且被广泛传播。员工能够充分描述这项倡议的各种预期益处。
- b变革的益处虽然有证据支持和被利益相关者认可，但是没有立即显现或被广泛传播。
- c变革的益处虽然有证据支持，但是未被利益相关者普遍认可，也没有立即显现或被广泛传播。
- d变革的益处没有证据支持或被利益相关者认可，也没有被广泛传播或立即显现。

**3改进流程的适应性**

问题描述

·新流程能否克服组织内部压力，还是会被内部压力中断？

·变革是否能继续有效地满足持续的需求？

·变革是否需要特定的个人或小组、技术、资金等来维持？

·当上述因素被移除时，变革能否继续进行？

- a改进的流程能适应组织的其他变化，甚至支持它，如果特定的人或小组离开该变革项目，流程不会被中断。流程的重心将会不断地满足我们组织的改进需求。
- b改进的流程能适应组织的其他变化，甚至支持它，但如果特定的人或小组离开该变革项目，流程会被中断。流程的要素将会不断地满足我们组织的改进需求。
- c 新流程难以适应组织的其他变化；如果特定的人或小组离开项目，会导致流程中断。
- d如果有任何组织变化发生，新流程将无法适应；且如果特定的人或小组离开，会导致流程中断

**4监测体系的有效性**

问题描述

·该变革是否需要专门的监测体系去识别和测量改进情况？

·是否有反馈体系来强化益处和进展，并发起新的倡议或进行下一步行动？

·是否建立了相关机制，以在项目正式结束后继续监测进展？

·变革的结果是否被传播到了患者、员工、组织以及更广泛的医疗保健社区？

- a有一个能反馈变革影响的体系，它包括效益分析、监测进展和传播结果。该体系是为了项目在正式结束后能够继续实施而建立的。
- b有一个能反馈变革影响的体系，它包括效益分析、监测进展和传播结果。该体系不是为了项目在正式结束后能够继续实施而建立的。
- c有一个能反馈变革影响和监测进展的体系，然而这些信息仅在项目核心团队中传播。该体系不是为了项目在正式结束后能够继续实施而建立的。
- d只有一个非常不完整的体系来监测进展，且该体系会随着项目结束而终止。没有一个体系去传播结果。

**二、员工**

**5员工参与和接受了培训以维持变革**

问题描述

·员工是否在变革的创新、设计和实施中发挥了作用？

·他们从一开始就对变革提出了自己的想法？

·是否有培训和形成识别技能和知识差距的基础设施，员工是否接受过教育和培训以推动变革？

- a员工从变革的初始阶段就参与其中。他们帮助识别任何技能差距，并且能够获得培训和成长，因此他们对新的工作方式充满信心并能够胜任它。
- b员工从变革的初始阶段就参与其中并帮助识别技能的差距，但他们没有接受过新工作方式的培训或没有在新的工作方式中得到成长。
- c员工从变革的初始阶段就没有参与其中，但他们接受了新工作方式的培训。
- d员工从变革的初始阶段就没有参与其中，也没有接受过新工作方式的培训或没有在新的工作方式中得到成长。

**6员工维持变革的行为**

问题描述

·在整个变革过程中，员工是否被鼓励表达，是否能够定期表达自己的想法，他们的意见是否被采纳？

·员工是否认为变革是他们想要在日后保留的、更好的工作方式？

·员工是否接受过培训并被授权根据他们的想法开展小规模试验（PDSA），以观察是否有其他需要改进的地方？

- a员工能够定期分享他们的想法，且其中的一些想法已经在项目期间被采纳。他们认为变革是一种更好的工作方式，且他们已经被授权开展小规模试验（计划、执行、研究、行动）。
- b员工能够定期分享他们的想法，且其中的一些想法已经在项目期间被采纳。他们认为变革是一种更好的工作方式，但他们没有被授权开展小规模试验（计划、执行、研究、行动）。
- c员工能够定期分享他们的想法，但没有任何想法在项目期间被采纳。他们不认为变革是一种更好的工作方式，也没有被授权开展小规模试验（计划、执行、研究、行动）。
- d员工觉得他们无法分享自己的想法。他们不认为变革是一种更好的工作方式，也没有被授权开展小规模试验（计划、执行、研究、行动）。

**7组织高层领导的参与和支持**

问题描述

·高级领导是否值得信赖、有影响力、受尊重和使人信服？

·他们是否参与了这一倡议，他们是否理解并推动该倡议？

·他们是否受到同行的尊重，他们能否影响其他人加入？

·他们是否承担起帮助克服障碍因素的个人责任，他们是否花费时间提供帮助以确保变革的成功？

- a组织领导高度参与且他们对变革流程的支持是显而易见的。他们会利用自己的影响力去传播变革的益处，和克服任何障碍。员工会定期与领导分享信息，且积极寻求领导的建议。
- b组织领导高度参与且他们对变革流程的支持是显而易见的。他们会利用自己的影响力去传播变革的益处，和克服任何障碍。但员工通常不会与领导分享信息，也不会向领导寻求建议。
- c组织领导在一定程度上参与了变革流程，但他们对变革流程的支持并不显著。他们会利用自己的影响力去传播变革的益处，但如果事情变得困难，就不能依靠他们克服任何障碍。员工一般不会与领导分享信息，也不会向领导寻求建议。
- d组织领导不参与或看不到他们对变革流程的支持。他们不会利用自己的影响力去传播变革的益处或克服任何障碍。员工一般不会与领导分享信息，也不会向领导寻求建议。

**8临床领导的参与和支持**

问题描述

·临床领导是否值得信任、有影响力、受尊重和使人信服？

·他们是否参与了这一倡议，他们是否理解并推动该倡议？

·他们是否受到同行的尊重，他们能否影响其他人加入？

·他们是否承担起帮助克服障碍因素的个人责任，他们是否花费时间提供帮助以确保变革的成功？

- a临床领导高度参与且他们对变革流程的支持是显而易见的。他们会利用自己的影响力来传播变革的益处，并克服任何障碍。员工会定期与临床领导分享信息，并积极向临床领导寻求建议。
- b临床领导高度参与且他们对变革流程的支持是显而易见的。他们会利用自己的影响力去传播变革的益处，并克服任何障碍。但员工通常不会与临床领导人员分享信息，也不会向临床领导寻求建议。
- c临床领导在一定程度上参与了变革流程，但他们对变革流程的支持并不显著。他们会利用自己的影响力去传播变革的益处，但如果事情变得困难，就不能依靠他们克服任何障碍。员工一般不会与临床领导分享信息，也不会向领导寻求建议。
- d临床领导不参与或看不到他们对变革流程的支持。他们不会利用自己的影响力去传播变革的益处或克服任何障碍。员工一般不会与临床领导分享信息，也不会向临床领导寻求建议。

**三、组织**

**9与组织的战略目标和文化相符**

问题描述

·变革的目标是否明确并得到公认？

·它们是否对组织的整体战略目标有明显贡献？

·变革对组织及其领导层是否重要？

·组织之前是否成功维持过变革？

- a变革的目标明确，并得到了普遍认同。它们与组织为了改进的战略目标一致并能够支持该目标。该组织之前有成功维持过变革，并且有一种“能做”的文化。
- b变革的目标明确，并得到了普遍认同。它们与组织为了改进的战略目标一致并能够支持该战略目标。该组织之前没有成功维持过变革，也没有“能做”的文化。
- c变革的目标明确，并得到了普遍认同。它们与组织的战略没有关联，所以我们不知道变革的目标是否支持组织任何为了改进的目标。该组织之前没有成功维持过变革，也没有“能做”的文化。
- d变革的目标并不明确，也没有得到普遍认同。它们与组织的战略没有关联，所以我们不知道变革的目标是否支持组织任何为了改进的目标。该组织之前没有成功维持过变革，也没有“能做”的文化。

**10基础建设**

问题描述

·员工是否接受过充分的培训并能胜任新的工作方式？

·是否有足够的设施和设备来支持新流程？

·新的要求是否被写入工作说明？

·是否有支持新工作方式的政策和程序？

·是否有适当的沟通系统？

- a员工对新的工作方式充满信心并且接受了培训。工作说明、政策和流程能够体现新的工作方式，且沟通系统已就位；设施和设备都适合维持新的变革。
- b员工对新的工作方式充满信心并且接受了培训。然而工作说明、政策和流程不能体现新的工作方式，一些沟通系统已就位；设施和设备都适合维持新的变革。
- c员工对新的工作方式充满信心并且接受了培训。然而工作说明、政策和流程不能体现新的工作方式，且没有能充分支持新的工作方式的沟通系统；设施和设备不适合维持新的变革。
- d员工没有接受过新流程的培训并且对新的工作方式没有信心。工作说明、政策和流程不能体现新的工作方式，且没有能充分支持新工作方式的沟通系统；设施和设备不适合维持新的变革。
